# Supplementary material for: Horizontal seed dispersal by dung beetles reduced seed and seedling clumping, but did not increase short-term seedling establishment
Source: PLoS One. 2019 Oct 24;14(10):e0224366. doi: 10.1371/journal.pone.0224366 (PMC6812793; doi:10.1371/journal.pone.0224366)
Supplement: S1 Table — Beetles captured using 10 pitfall traps each baited with 50 g of fresh domestic pig dung and opened during 48 hours. Sampling was conducted in April, September and October 2016. Information about dung relocation behavior (tunneler ‘T’, roller ‘R’, dweller ‘D’), diet (predominantly feces ‘F’ or carrion ‘C’), and body measurements (dry weight, body length) are from Díaz & Favila [2009. Escarabajos coprófagos y necrófagos (Scarabaeidae, Silphidae y Trogidae) de la reserva de la biosfera Los Tuxtlas, México. Memorias VIII Reunión Latinoamericana de Scarabaeidología (Coleoptera: Scarabaeoidea). Pp. 34. Xalapa, Veracruz]. *Eurysternus has a unique behavior in which dung is not relocated; unlike typical rollers, feeding takes place directly in the dung source, but unlike typical dwellers, dung balls are made for nesting and are lightly covered by soil near the dung source [Halffter, G., & Edmonds, W. D. 1982. The nesting behavior of dung beetles (Scarabaeinae). An ecological and evolutive approach (Man and the Biosphere Program, Publication 10). Instituto de Ecología, Mexico City]. (DOCX) [file pone.0224366.s001.docx]

**S1 Table. Dung beetles captured in the Los Tuxtlas Biological Station, Veracruz, Mexico.**

| **Species** | **Abundance** | | | | **Behavior** | | | **Diet** | | **Body measurements** | | |
| --- | --- | --- | --- | --- | --- | --- | --- | --- | --- | --- | --- | --- |
|  | **Apr.** | **Sept.** | **Oct.** | **Total** | **T** | **R** | **D** | **D** | **C** | **Weight (mg)** | | **Length (mm)** |
| *Ateuchus illaesum* Harold, 1869 | 53 | 65 | 43 | 161 | x |  |  | x |  | 11 | | 7 |
| *Bdelyropsis newtoni* Howden, 1971 | 0 | 1 | 6 | 7 | x |  |  | x |  | 14 | | 4 |
| *Canthidium centrale* Boucomont, 1928 | 5 | 9 | 5 | 19 | x |  |  | x |  | 18 | | 8 |
| *Canthon cyanellus cyanellus* LeConte, 1859 | 5 | 1 | 0 | 6 |  | x |  |  | x | 23 | | 8 |
| *Canthon femoralis* (Chevrolat, 1834) | 5 | 5 | 0 | 10 |  | x |  | x |  | 16 | | 7 |
| *Canthon vazquezae* Martínez, Halffter & Halffter, 1964 | 2 | 6 | 1 | 9 |  | x |  | x |  | 12 | | 6 |
| *Copris laeviceps* Harold, 1869 | 90 | 52 | 50 | 192 | x |  |  | x |  | 25 | | 10 |
| *Deltochilum pseudoparile* Paulian, 1938 | 3 | 2 | 3 | 8 |  | x |  |  | x | 38 | | 10 |
| *Deltochilum sublaeve* Bates, 1887 | 0 | 2 | 0 | 2 |  | x |  |  | x | 415 | | 23 |
| *Dichotomius satanas* (Harold, 1867) | 42 | 101 | 7 | 150 | x |  |  | x |  | 173 | | 20 |
| *Eurysternus angustulus* Harold, 1869 | 4 | 0 | 0 | 4 |  |  | x* | x |  | 23 | | 9 |
| *Eurysternus mexicanus* Harold, 1869 | 7 | 2 | 0 | 9 |  |  | x* | x |  | 22 | | 11 |
| *Onthophagus batesi* Howden & Cartwright, 1963 | 44 | 1 | 4 | 49 | x |  |  | x |  | 9 | | 7 |
| *Onthophagus incensus* Say, 1835 | 97 | 6 | 137 | 240 | x |  |  | x |  | 8 | | 7 |
| *Onthophagus rhinolophus* Harold, 1869 | 24 | 16 | 30 | 70 | x |  |  | x |  | 9 | | 7 |
| *Phanaeus endymion* Harold, 1863 | 0 | 0 | 2 | 2 | x |  |  | x |  | 103 | | 15 |
| *Uroxys boneti* Pereira & Halffter, 1961 | 7 | 4 | 3 | 14 | x |  |  | x |  | 1 | | 3 |
| *Uroxys platypyga* Howden & Young, 1981 | 2 | 2 | 0 | 4 | x |  |  | x |  | 1 | | 3 |
| **Total number of individuals** | **390** | **275** | **291** | **956** | **908** | **35** | **13** | **940** | **16** |  | |  |
| **Total number of species** | **15** | **16** | **12** |  | **11** | **5** | **2** | **15** | **3** |  |  | |
