# Supplementary material for: Horizontal seed dispersal by dung beetles reduced seed and seedling clumping, but did not increase short-term seedling establishment
Source: PLoS One. 2019 Oct 24;14(10):e0224366. doi: 10.1371/journal.pone.0224366 (PMC6812793; doi:10.1371/journal.pone.0224366)
Supplement: S1 Fig — (A) 50 g of fresh domestic pig dung used in plots with dung added; dung was divided into 4 equal portions, each containing 5 seeds of either Bursera simaruba or Poulsenia armata; (B) seeds inside the plots with no dung added; seeds were placed directly on the soil surface (as indicated by the red arrows); (C and D) experimental seeds of Poulsenia thread-marked with a 30 cm-long fishing line in Experiment 1 (for Experiment 2, seeds were not thread-marked); (E) mosquito netting excluded dung beetles from control plots during the first 48 h (both experiments), and also excluded seed rain and seed/seedling predators from all plots after the first 48 h (Experiment 2); (F) plot with dung beetle activity after 48h of having placed the dung piles containing seeds; no dung remains visible on the soil surface; (G) grid (2 cm) used to map the location of each seed (Experiment 1) and seedling (Experiment 2), to calculate the nearest neighbor index; (H and I) seedlings of Bursera and Poulsenia, respectively, establishing inside plots from experimental seeds. (DOCX) [file pone.0224366.s002.docx]

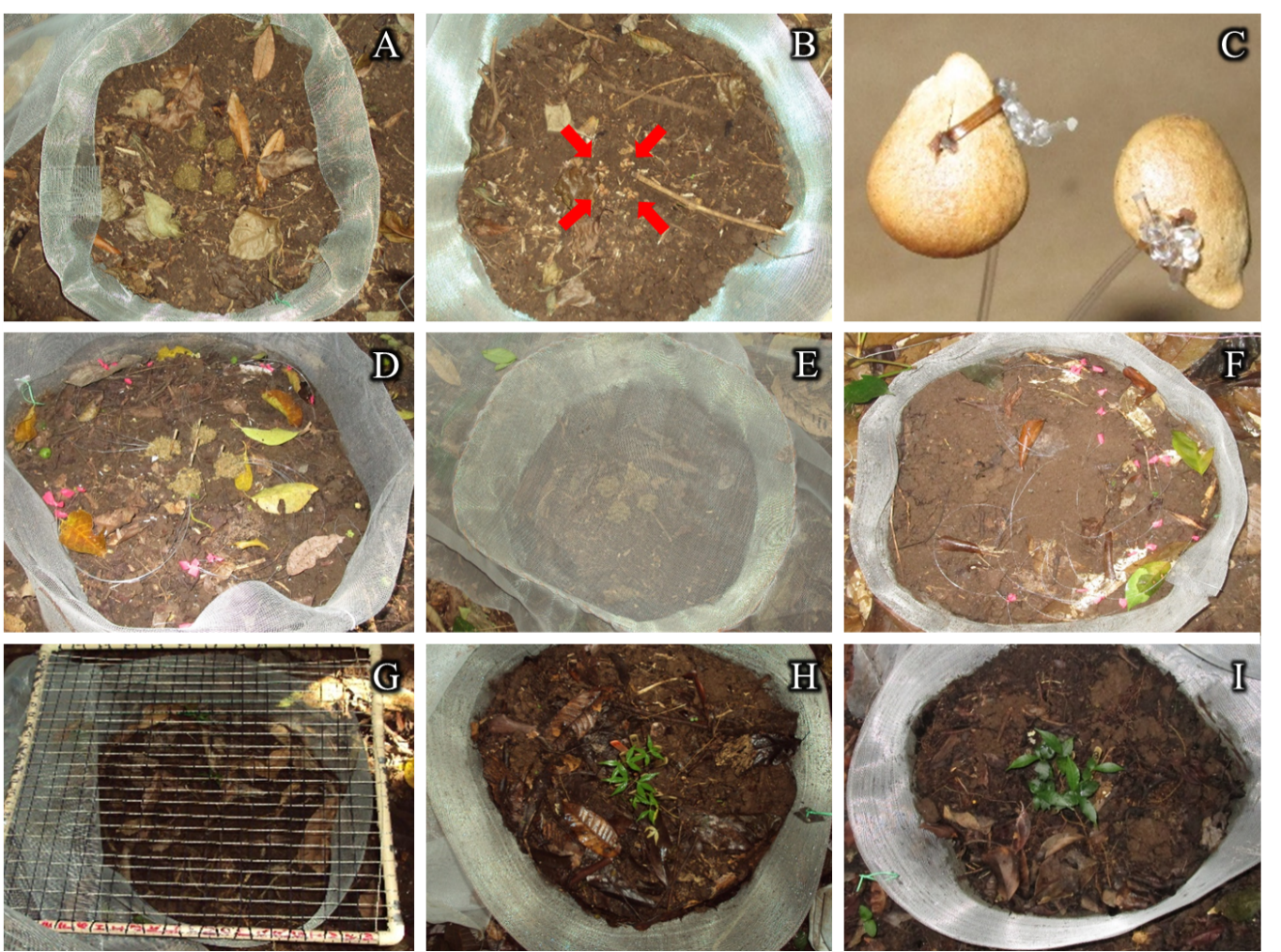


**S1 Fig. Methodological details of the two experiments carried out to assess the effects of dung beetle activity on the spatial distribution of seeds (Experiment 1) and seedlings (Experiment 2), and on the probability of seedling establishment (Experiment 2).**
