# Supplementary figures and images for: Horizontal seed dispersal by dung beetles reduced seed and seedling clumping, but did not increase short-term seedling establishment
Source: PLoS One. 2019 Oct 24;14(10):e0224366. doi: 10.1371/journal.pone.0224366 (PMC6812793; doi:10.1371/journal.pone.0224366)

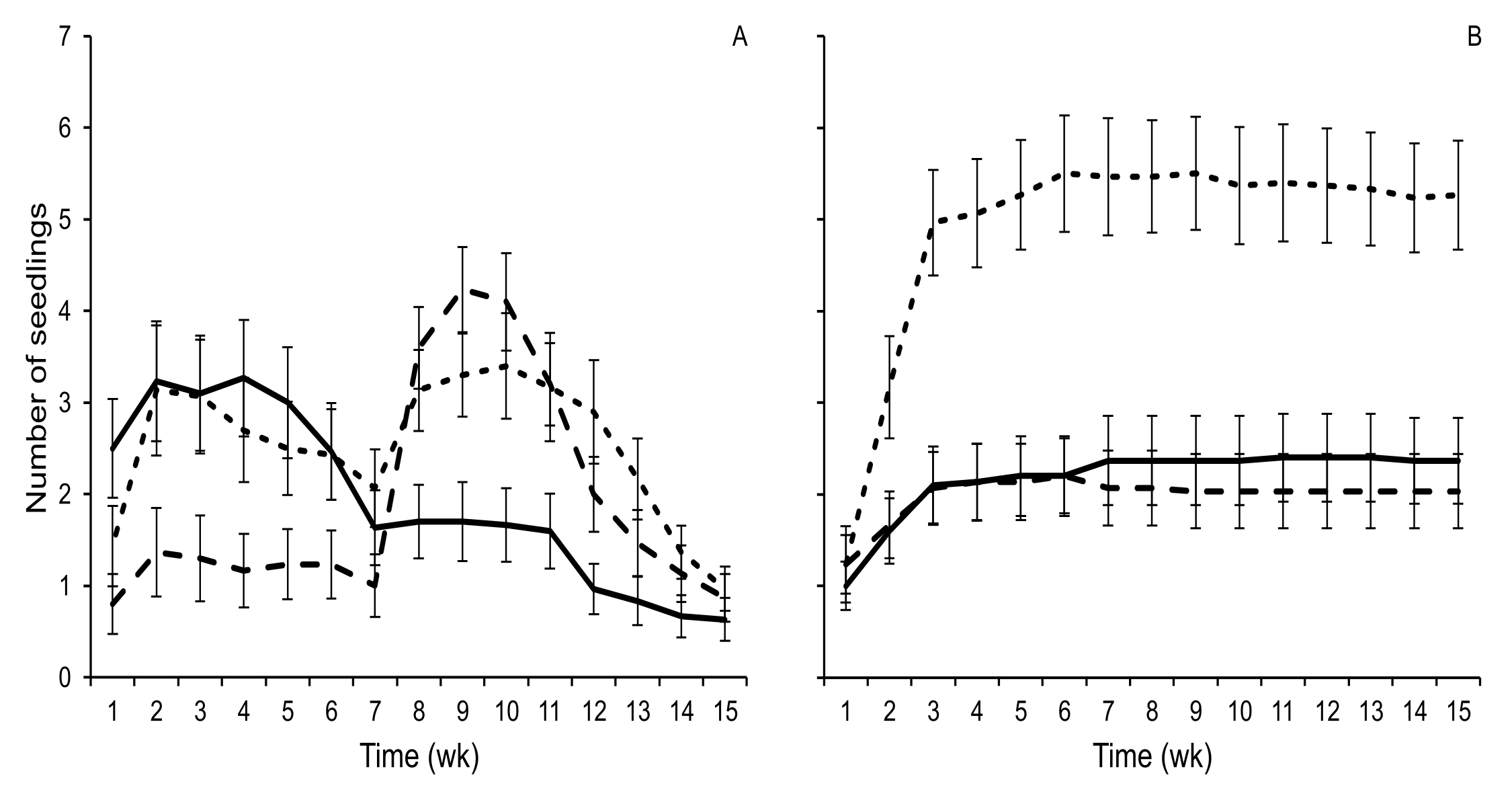

Supplement: S2 Fig — Bursera simaruba (A) and Poulsenia armata (B) seedlings established in 50-cm-diam plots (N = 30 for each species-treatment level) with three treatment levels: 50 g of dung and beetle access (black continuous line), 50 g of dung and beetle exclusion (black dashed line), and with no dung or beetles (black dotted line). In each plot of the first two treatment levels 20 seeds were mixed in the dung, in the last treatment level seeds were placed on the soil surface. Error bars represent ± 1 SE. (TIF) [file pone.0224366.s003.tif]

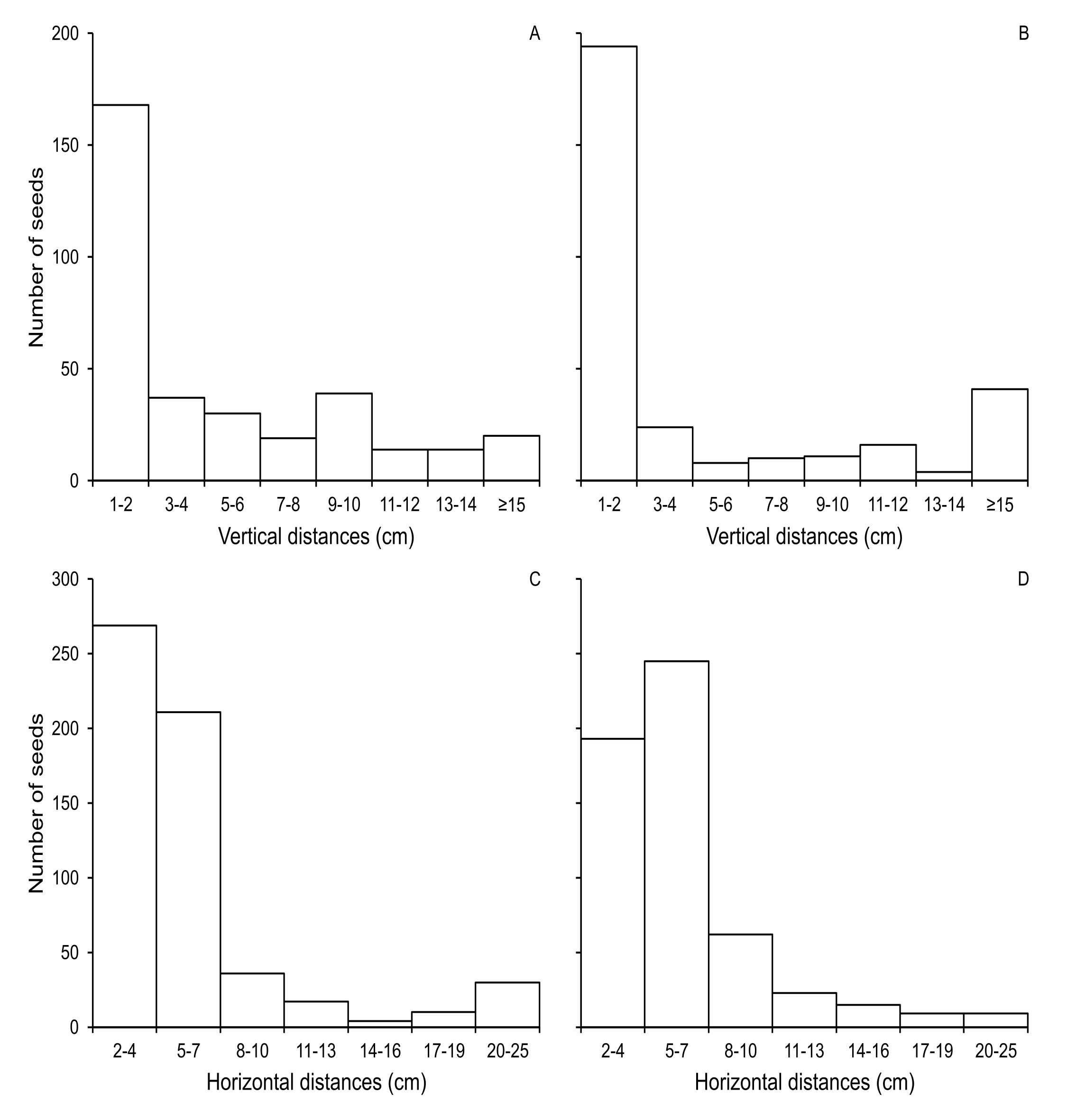

Supplement: S3 Fig — Vertical (A and B) and horizontal (C and D) dispersal distances for experimental seeds that were secondarily dispersed by dung beetles (seeds that remained in their original position were not dispersed and thus were not included in these graphs). Seed species are Bursera simaruba (A and C) and Poulsenia armata (B and D). Dung beetle activity was restricted to circular plots 25 cm in radius; inside each plot 50 g of fresh pig dung containing 20 seeds of one species was placed. (TIF) [file pone.0224366.s004.tif]

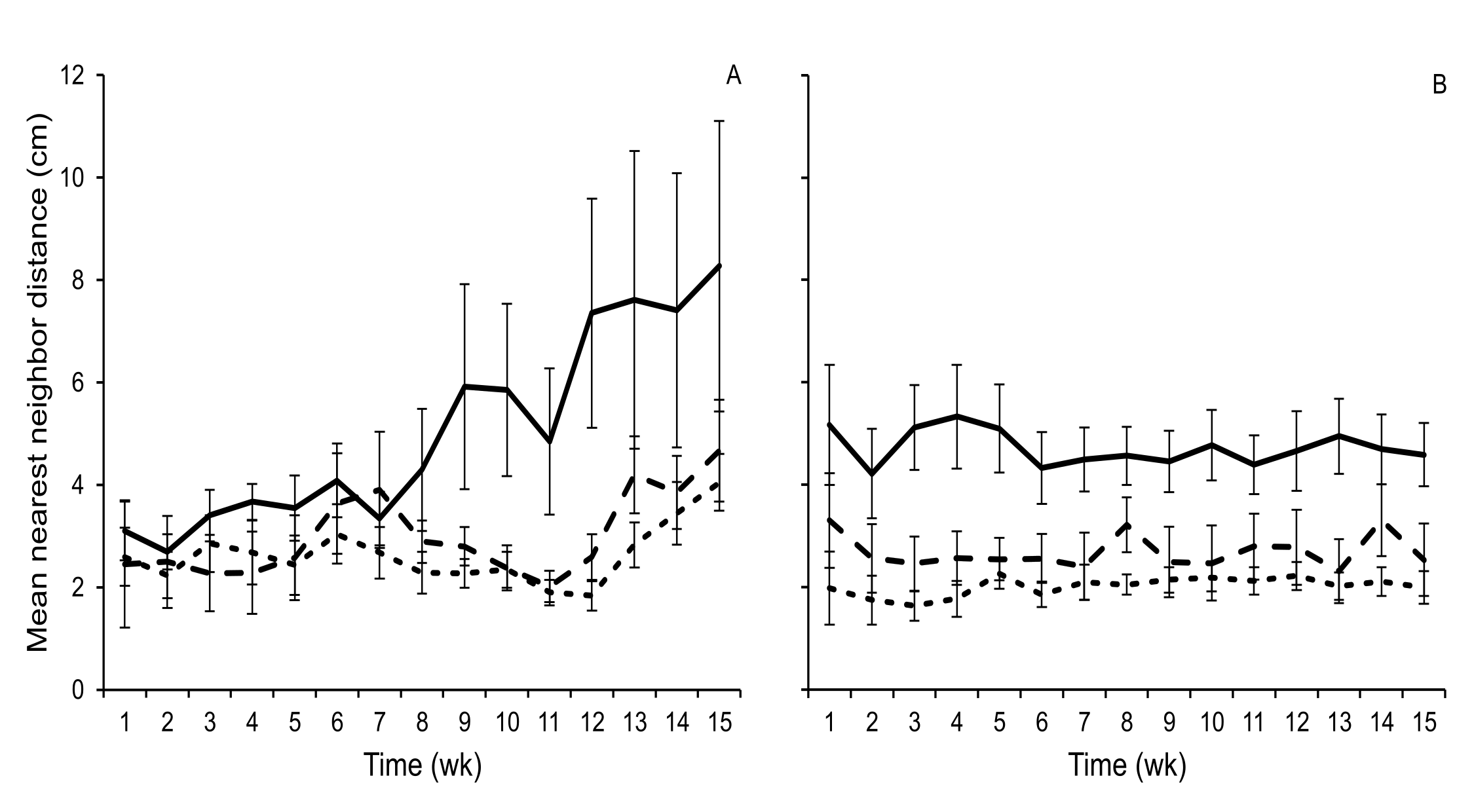

Supplement: S4 Fig — Distance for seedlings of Bursera simaruba (A) and Poulsenia armata (B) over 15 weeks in plots (N = 30 for each species-treatment level) with three treatment levels: 50 g of dung and beetle access (black continuous line), 50 g of dung and beetle exclusion (black dashed line), and with no dung or beetles (black dotted line). Error bars represent ± 1 SE. (TIF) [file pone.0224366.s005.tif]
