# Supplementary material for: Horizontal seed dispersal by dung beetles reduced seed and seedling clumping, but did not increase short-term seedling establishment
Source: PLoS One. 2019 Oct 24;14(10):e0224366. doi: 10.1371/journal.pone.0224366 (PMC6812793; doi:10.1371/journal.pone.0224366)
Supplement: S1 Appendix — (DOCX) [file pone.0224366.s006.docx]

**S1 Appendix. Methods and results of dung beetle sampling in the Los Tuxtlas Biological Station, Veracruz, Mexico.**

To determine the structure and composition of the dung beetle assemblage responsible for the results obtained in our two field experiments, we sampled dung beetles. We used 10 pitfall traps (separated from each other by ≥100 m), each baited with 50 g of fresh domestic pig dung, i.e., the same type of dung we used in our experiments. The 10 traps were deployed three times (without changing their location): in April, September and October 2016, and opened for 48 h every time. All individuals were counted and identified in the Laboratorio de Ecoetología at the Instituto de Ecología A.C., Xalapa,Veracruz, Mexico. Also, we used a dung beetle collection with specimens collected from LTBS and Los Tuxtlas region.

Overall, we captured a total of 956 dung beetles of 18 species (S1 Table); at the trap level, we captured an average of 32 (SD: ± 13) individuals and 7 (± 2) species in each 48-h sampling. According to their functional traits, 11 species were classified as tunnelers, 5 as rollers, and 2 as dwellers; 15 species were considered mainly coprophagous, while 3 species were considered primarily necrophagous. Beetle body length ranged from 3 to 23 mm, with 33% of all species and 38% of all individuals captured being relatively large (body length ≥10 mm, S1 Table).
