# Supplementary material for: Horizontal seed dispersal by dung beetles reduced seed and seedling clumping, but did not increase short-term seedling establishment
Source: PLoS One. 2019 Oct 24;14(10):e0224366. doi: 10.1371/journal.pone.0224366 (PMC6812793; doi:10.1371/journal.pone.0224366)
Supplement: Attachment — Submitted filename: Response to Reviewers.docx [file pone.0224366.s022.docx]

Dear Dr. Guillermo C. Amico,

We have read and addressed all the comments made by Dr. Laurence Culot (Reviewer #3); please see our responses (in CAPITAL LETTERS) to the comments detailed below. Once again, we thank you and the referees for all comments done to improve our manuscript.

Sincerely yours,

L.A. Urrea-Galeano and E. Andresen, on behalf of all co-authors

Reviewer #1: (No Response)

Reviewer #2: This revised version of the manuscript has improved considerably. The methods are written down in a way it is possible to reproduce the experiment, and I really appreciate the pictures that are included as an appendix. The whole text is well written, and the images are clear. All suggestions and corrections I proposed were addressed properly, and questions were answered. After carefully reading the manuscript, inspecting the graphs and appendices, no new issues raised, so I would advise to publish this paper. THANK YOU!

Reviewer #3: You responded satisfactorily to most of my comments and I think that the manuscript has been well improved. THANK YOU!

I still think that you can expect a different secondary seed dispersal activity between seasons, and therefore between the experiment 1 and 2. You may have a low proportion of rollers, but the tunnelers also influence the burial depth, which consequently can affect the probability of seedling emergence. This difference does not discredit your work, but I think that a mention of this possible bias should be mentioned in the discussion. THANKS FOR YOUR OBSERVATION, WE AGREE. WE HAVE MENTIONED THIS ISSUE IN TWO PARTS IN THE DISCUSSION. IN LINES 401-406 WE HAVE WRITTEN THIS: “However, it could also be that seasonal differences in dung beetle assemblages caused a higher proportion of seeds to be buried and/or buried more deeply in Experiment 2, which was setup in April-June, than in Experiment 1, which was done in October. When we sampled dung beetles, we found higher abundances, particularly of large beetles (≥ 10 cm body length), in April than in October (S1 Table).” ALSO, IN LINES 478-483 WE HAVE ADDED THIS TEXT: “For example, differences between species could be due to seasonality in dung beetle activity, since the experiment for Bursera was started in April and the one with Poulsenia in June, a drier and a rainier month, respectively. Secondary seed dispersal by dung beetles has been shown to be affected by seasonality [7], not only because dung beetle assemblages vary seasonally [10], but also because in rainier months softer soils might favor deeper seed burial [21], which in turn might hinder seedling emergence.” FINALLY, IN S1 TABLE WE HAVE ADDED INFORMATION ON THE NUMBER OF INDIVIDUALS CAPTURED IN EACH OF THE SAMPLINGS, SO THAT THE POTENTIAL EFFECT OF SEASONALITY CAN BE BETTER APPRECIATED.

Please find a few minor comments based on the reading of this new version of the manuscript:

L197-198: What do you mean by “conservative” in this case. The restricted area of your experiments can bias the results towards an underestimation of the horizontal distances. Is it what you mean? If so, I suggest to rephrase. If not, please clarify or delete the sentence and keep this information for the discussion. YES, UNDERESTIMATION IS WHAT WE MEAN; WE HAVE REWRITTEN THE SENTENCE FOR CLARITY (LINES 197-198). NOW IT READS: “While this allowed us to find most seeds, it probably caused some underestimation of horizontal distances (see Discussion).”

L205: I suggest writing “one of the driest months” DONE (LINE 205)

L2016: I suggest writing “the beginning of the rainy season” DONE (LINE 206)
